# Abietane Diterpenoids Isolated from *Torreya nucifera* Disrupt Replication of Influenza Virus by Blocking the Phosphatidylinositol-3-Kinase (PI3K)-Akt and ERK Signaling Pathway

**DOI:** 10.3390/cimb45030147

**Published:** 2023-03-09

**Authors:** Jaehoon Bae, Hyung-Jun Kwon, Ji Sun Park, Jinseok Jung, Young Bae Ryu, Woo Sik Kim, Ju Huck Lee, Jae-Ho Jeong, Jae Sung Lim, Woo Song Lee, Su-Jin Park

**Affiliations:** 1 Functional Biomaterial Research Center, Korea Research Institute of Bioscience and Biotechnology, 181 Ipsin-gil, Jeongeup-si 56212, Republic of Korea; 2 Biological Resource Center, Korea Research Institute of Bioscience and Biotechnology, 181 Ipsin-gil, Jeongeup-si 56212, Republic of Korea; 3 Department of Microbiology, Chonnam National University Medical School, Gwangju 61468, Republic of Korea; 4 College of Pharmacy, Chonnam National University, Gwangju 61186, Republic of Korea

**Keywords:** abietane diterpenoids, *Torreya nucifera*, influenza virus, PI3K-Akt, viral replication

## Abstract

Although vaccines and antiviral drugs are available, influenza viruses continue to pose a significant threat to vulnerable populations globally. With the emergence of drug-resistant strains, there is a growing need for novel antiviral therapeutic approaches. We found that 18-hydroxyferruginol (**1**) and 18-oxoferruginol (**2**) isolated from *Torreya nucifera* exhibited strong anti-influenza activity, with 50% inhibitory concentration values of 13.6 and 18.3 μM against H1N1, 12.8 and 10.8 μM against H9N2, and 29.2 μM (only compound **2**) against H3N2 in the post-treatment assay, respectively. During the viral replication stages, the two compounds demonstrated stronger inhibition of viral RNA and protein in the late stages (12–18 h) than in the early stages (3–6 h). Moreover, both compounds inhibited PI3K-Akt signaling, which participates in viral replication during the later stages of infection. The ERK signaling pathway is also related to viral replication and was substantially inhibited by the two compounds. In particular, the inhibition of PI3K-Akt signaling by these compounds inhibited viral replication by sabotaging influenza ribonucleoprotein nucleus-to-cytoplasm export. These data indicate that compounds **1** and **2** could potentially reduce viral RNA and viral protein levels by inhibiting the PI3K-Akt signaling pathway. Our results suggest that abietane diterpenoids isolated from *T. nucifera* may be potent antiviral candidates for new influenza therapies.

## 1. Introduction

Influenza viruses are responsible for seasonal flu epidemics and cause acute contagious respiratory infections. In particular, influenza A viruses caused three pandemics in the 20th century and are known to be transmitted to other species [1,2]. More recently, the human influenza outbreak of the swine-origin A/H1N1 strain in 2009 has become a serious public health concern around the world [3,4]. Three viral proteins, neuraminidase (NA) (oseltamivir, zanamivir, and peramivir), the M2 ion channel protein (amantadine and rimantadine), and polymerase (baloxavir marboxil), are targets for FDA-approved influenza antiviral drugs [5]. Unfortunately, there is now widespread resistance to all of these drug classes [6,7]. Combined with the limited number of viral drug targets for influenza virus, this creates concern for the development of new influenza therapies.

Virus survival relies upon the evolution of strategies that modulate host cell signaling pathways, particularly those governing apoptosis and cell survival [8]. The replication of influenza viruses also depends on the host cellular machinery involved in cell survival, such as Raf/MEK/extracellular signal-regulated kinase (ERK) and phosphatidylinositol 3-kinase (PI3K)-Akt pathways [9]. Among them, PI3K is a key modulator of many signal transduction pathways and acts on several downstream effectors, including Akt and ERK, to regulate a diverse range of cellular events [10]. Initially, these pathways were thought to have an antiviral effect [11]. This signaling is activated by the binding of viral nonstructural protein 1 (NS1) to the p85β regulatory subunit of PI3K [12,13]. It has also been reported that ERK phosphorylation supports influenza virus replication [14,15,16]. In other words, at each stage of infection, influenza virus can differentially stimulate cellular signaling cascades, some of which are important components of antiviral immunity, whereas others are essential for sufficient viral replication. Therefore, blocking viral-infection-induced cellular signal transduction (including PI3K-Akt and ERK signals) required for viral replication might be an effective strategy for developing new antiviral therapeutics.

*Torreya nucifera* (*T. nucifera*) is a Taxaceae tree found in snowy areas near the Sea of Jeju Island in Korea and has been used in traditional Asian medicine as a remedy for stomachaches, hemorrhoids, and rheumatoid arthritis [17]. Various abietane diterpenoids have been isolated from the leaves of *T. nucifera* and from suspension-cultured cells of *T. nucifera var. radicans*, but the bioactivity of these diterpenoids has not been evaluated [18,19]. The abietane diterpenoids isolated from rosemary (*Rosmarinus officinalis* L.) were shown to have antioxidant activity [20]. In general, the abietane diterpenoid ferruginol is known to possess a variety of biological activities, including anti-SARS-CoV 3CLpro [21], anti-bacterial [22], cardioactive [23], and anticancer activities [22]. Notably, ferruginol was reported to have anticancer activity via its inhibition of Ras/PI3K, protein tyrosine phosphatase, and protein kinases [22]. Interestingly, it was reported that influenza virus replication was inhibited by blocking the PI3K-Akt signaling pathway with the traditional Chinese herbal medicine Ko-Ken Tang [9]. However, based on our knowledge, it is unclear whether abietane diterpenoids isolated from *T. nucifera* show anti-influenza-virus activity. In this study, we examined the antiviral effects and their downstream mechanisms (PI3K-Akt and ERK signaling pathway) of abietane diterpenoids.

## 2. Materials and Methods

### 2.1. Materials

In October 2003, the leaves of T. nucifera were collected from Jeju Island, Republic of Korea. A voucher specimen was deposited in the author’s laboratory at the Korea Research Institute of Bioscience and Biotechnology (KRIBB) [21].

### 2.2. Cell and Viruses

Madin–Darby canine kidney (MDCK) cells were obtained from ATCC CCL-3 (Manassas, VA, USA) and grown in Eagle’s minimum essential medium (EMEM) supplemented with 10% fetal bovine serum (FBS), 100 U/mL penicillin, and 100 μg/mL streptomycin at 37 °C under 5% CO_2_. Influenza strains A/PR/8/34 (H1N1) (ATCC VR-1469), A/Chicken/Korea/MS96/96 (H9N2), and A/Hong Kong/8/68 (H3N2) (ATCC VR-544) were propagated in MDCK cells in the presence of 10 μg/mL trypsin (GIBCO Invitrogen Corporation, CA, USA). MDCK cells were grown in 96-well plates at 1 × 10^5^ cells/well for 24 h. The media in the plates were replaced with those containing the serially diluted extract or compounds (1–200 μg/mL or μM) and incubated at 37 °C under a 5% CO_2_ atmosphere for 72 h. The MTT analysis was used to evaluate cell viability according to the manufacturer’s instructions.

### 2.3. Time Course (Time-of-Addition) Antiviral Assays

We used a modified protocol based on Kwon et al. [24] to evaluate the antiviral efficacy of the drugs in vitro. In brief, MDCK cells were grown in 96-well plates at 1 × 10^5^ cells/well for 24 h. In a pre-treatment assay, before virus inoculation, the extract or compounds at different concentrations were added to the cells and incubated for 12 h. Then, the extract and compounds were removed, and the MDCK cells were washed 2 times with PBS. Influenza virus at 0.001 MOI was inoculated into the MDCK cells for 1 h with occasional rocking. The supernatant was removed and replaced by EMEM containing 10 μg/mL trypsin. In a simultaneous treatment assay, various concentrations of extract or compounds were mixed with the virus at 0.001 MOI and incubated at 4 °C for 1 h. The mixtures were inoculated onto near-confluent MDCK cell monolayers (1 × 10^5^ cells/well) for 1 h with occasional rocking. The solution was removed and replaced by EMEM containing 10 μg/mL trypsin. In a post-treatment assay, influenza virus at 0.001 MOI was inoculated onto near-confluent MDCK cell monolayers for 1 h with occasional rocking. The media were removed and replaced by EMEM containing 10 μg/mL trypsin and extract or compounds at different concentrations. The extract and compounds were assayed for virus inhibition in triplicate. After 72 h incubation in all antiviral assays, 0.034% neutral red was added to each well and incubated for 2 h at 35 °C in the dark. The neutral red solution was removed, and the cells were washed with PBS (pH 7.4). Destaining solution (containing 1% glacial acetic acid, 49% H_2_O, and 50% ethanol) was added to each well. The plates were incubated in the dark for 15 min at room temperature. Absorbance was read at 540 nm using a microplate reader. The 50% inhibitory concentration (IC_50_) value was calculated by regression analysis. A selective index (SI) was calculated using the following formula: SI = 50% cytotoxicity concentration (CC_50_)/IC_50_.

### 2.4. Chemiluminescent Neuraminidase Inhibition (NAI) Assay

The chemiluminescent NAI assay was conducted using the commercially available NA-Star Kit (Applied Biosystems, CA, USA) consisting of NA-Star buffer (26 mM morpholineethanesulfonic acid, 4 mM CaCl_2_ [pH 6.0]), NA-Star substrate, NA-Star accelerator, and 96-well solid white plates. Serial two-fold dilutions of each working NA inhibitor were prepared in NA-Star buffer. The protocol was performed essentially as recommended by the manufacturer.

### 2.5. Quantitative Real-Time PCR (qRT-PCR)

MDCK cells were grown to about 90% confluence, infected with influenza virus at 0.001 MOI and cultured in the presence of 18-hydroxyferruginol (**1**), 18-oxoferruginol (**2**), and oseltamivir. The infected/untreated cells were cultured in the presence of 0.5% DMSO. The medium was removed after 3, 6, 12, and 18 h. Cells were scraped off, washed twice with phosphate buffer saline (PBS), and collected by centrifugation (500× *g* for 3 min). The total RNA was isolated using the Qiagen RNeasy mini kit (QIAGEN, Hilden, Germany) according to the manufacturer’s instructions. The specific primers were used for the reverse transcription of viral RNA (vRNA; M gene, 5′-CTTCTAACCGAGGTCGAAACGTA-3′ and 5′-GGTGACAGGATTGGTCTTGTCTTTA-3′; NP gene, 5′-AGRTAYTGGGCYATAAGRAC-3′ and 5′-CTTATTTCTTCGGAGACAATGC-3′). GAPDH was used as the internal control for cellular RNAs, with primer sequences of 5′-TCAACGGATTTGGCCGTATTGG-3′ and 5′-TGAAGGGGTCATTGATGGCG-3′. cDNA was synthesized from total RNA using the cDNA Master Mix (Applied Biosystems, CA, USA). qRT-PCR was conducted using 2 μL of cDNA and the Power SYBR Green PCR 2 X Master Mix (Applied Biosystems, CA, USA). qRT-PCR was conducted using the Step One Plus Real-time PCR System, and the data were analyzed with the StepOne software v2.1 (Applied Biosystems, CA, USA).

### 2.6. Western Blotting Analysis

MDCK cells were infected with H1N1, H9N2, or H3N2 at 0.001 MOI for 1 h and treated with either 0.5% DMSO, 50 μM LY294002, 50 μM PD98059, or 20 μM of either 18-hydroxyferruginol (**1**) or 18-oxoferruginol (**2**). Cells were lysed with lysis buffer (50 mM Tris-HCl (pH 7.5), 30 mM Na_4_P_2_O_7_, 1 mM pervanadate, 150 mM NaCl, 1% Triton X-100, 50 mM NaF, 1 mM ethylenediaminetetraacetic acid, 2 mg/mL aprotinin, and 1 mM phenylmethanesulfonyl fluoride), and approximately 50 μg of protein was loaded onto SDS-polyacrylamide gels and then electroblotted onto Trans-Blot nitrocellulose membranes. Primary antibodies were used at a 1:1000 dilution and incubated overnight at 4 °C. Anti-p-Akt and p-ERK1/2 antibodies were purchased from Cell Signaling Technology Inc. (Boston, MA, USA). Anti-p-MEK1/2, GAPDH, and influenza virus NS1 antibodies were purchased from Santa Cruz Biotechnology Inc. (Santa Cruz, CA, USA), and an anti-influenza NP antibody was purchased from Millipore (Bedford, MA, USA). After washing with Tween 20-PBS, blots were incubated with anti-rabbit HRP conjugates (secondary antibody) at a 1:10,000 dilution for 1 h. The band density was quantified using ImageJ software. All solvents were of analytical grade and obtained from Burdick & Jackson.

### 2.7. Confocal Fluorescence Imaging

MDCK cells were grown on a chamber slide, pre-coated with poly-L-lysine, and the monolayers were infected with influenza virus at 0.001 MOI for 1 h. The virus was removed and replaced by EMEM containing 10 μg/mL trypsin and each compound under test. All cells were cultured for 10 h at 37 °C in a 5% CO_2_ atmosphere and fixed in 4% paraformaldehyde solution for 15 min at room temperature. After washing, the cells were incubated at 37 °C for 1 h with an FITC-conjugated nucleoprotein (NP)-specific monoclonal antibody against influenza virus (Millipore, MA, USA) diluted at 1:50. After washing with PBS, cells were counterstained with 500 nM propidium iodide solution. Slides were mounted using the SlowFade Gold antifade reagent (Invitrogen, CA, USA). Confocal fluorescence imaging was performed using the Carl Zeiss LSM 510 META confocal microscope (Carl Zeiss Inc., Jena, Germany).

### 2.8. Statistical Analysis

All experiments were performed three times. Data are expressed as the mean ± SE. Statistical analysis was performed using the Sigma Plot Statistical Analysis software. Differences between group mean values were determined by one-way analysis of variance followed by a two-tailed Student’s *t*-test for unpaired samples, assuming equal variances.

## 3. Results

### 3.1. Inhibition of the Influenza Virus Life Cycle by 18-Hydroxyferruginol *(**1**)* and 18-Oxoferruginol *(**2**)*

**Figure 1:**
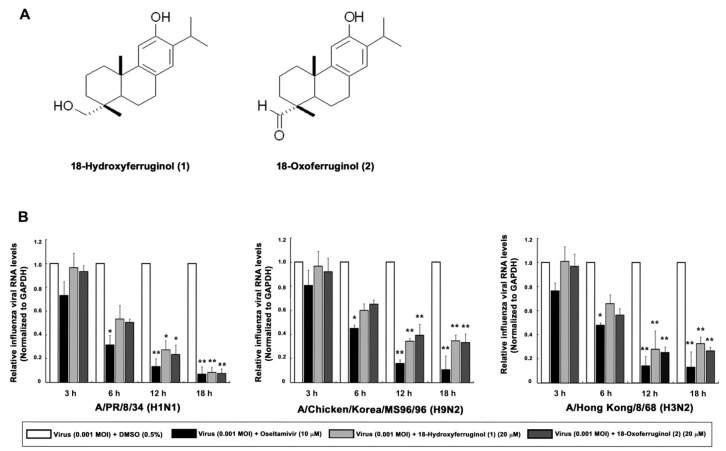
Effects of 18-hydroxyferruginol (**1**) and 18-oxoferruginol (**2**) on influenza viral RNA levels (M gene). (**A**) The structures of 18-hydroxyferruginol (**1**) and 18-oxoferruginol (**2**). (**B**) MDCK cells were infected with influenza viruses (0.001 MOI) for 1 h, viruses were removed, and cells were treated with 0.5% DMSO, 20 μM 18-hydroxyferruginol (**1**), 20 μM 18-oxoferruginol (**2**), or 10 μM oseltamivir for 3, 6, 12, and 18 h. Total RNA was extracted after virus infection, and the level of the M gene was measured. * *p* < 0.05; ** *p* < 0.01.

We show the structures of 18-hydroxyferruginol (**1**) and 18-oxoferruginol (**2**) (Figure 1A). Diterpenoid species compounds were isolated from the n-hexane fraction (Figure 1A). The cytotoxicity of the two isolated compounds was determined using the CC_50_ values, which were calculated from the MTT assay in MDCK cells. At a concentration of compounds **1** and **2** of <20 μM, no toxic effect was observed on MDCK cells. Subsequent experiments designed to evaluate the antiviral effect were carried out at the minimally toxic concentrations of the compounds (>98% cell viability).

To determine the viral replication stage at which the compounds exerted the maximum inhibitory effects, we performed time-of-addition experiments. Subsequently, 18-hydroxyferruginol (**1**) showed the most antiviral activity against A/PR/8/34 (H1N1) (IC_50_ = 13.6 μM, SI = 1.68) and A/Chicken/Korea/MS96/96 (H9N2) (IC_50_ = 12.8 μM, SI = 1.79) in the post-treatment assay. Notably, 18-oxoferruginol (**2**) exerted antiviral activity against all strains with the following rank order of potency: A/Chicken/Korea/MS96/96 (H9N2) (IC_50_ = 10.8 μM, SI = 3.6) > A/PR/8/34 (H1N1) (IC_50_ = 18.3 μM, SI = 2.13) > A/Hong Kong/8/68 (H3N2) (IC_50_ = 29.2 μM, SI = 1.33) (Table 1). These results demonstrate that compounds **1** and **2** inhibited influenza virus in the post-treatment assay and further indicate that the antiviral activity of these compounds differs according to the strain of the virus. Furthermore, chemiluminescence-based NAI assays showed little NA-inhibitory activity of the two compounds against the three strains (Table 2).

As influenza vRNA is synthesized in the early (3–6 h) and late stages (12–18 h), we tested the effects of these compounds on both of these synthesis stages in infected cells by qRT-PCR. We showed that influenza vRNA levels (M gene) were markedly decreased by 65.3–91.6% by compound **1** and 60.7–92.4% by compound **2** (20 μM) in all virus strains at 12 and 18 h compared with infected/untreated cells (0.5% DMSO) (Figure 1B). These results indicate that compounds **1** and **2** exert a stronger inhibitory effect on the late stage of viral replication than on the early stage.

### 3.2. Inhibition of Influenza-Virus-Induced Activation of the PI3K-Akt Signaling Pathway by 18-Hydroxyferruginol *(**1**)* and 18-Oxoferruginol *(**2**)*

**Figure 2:**
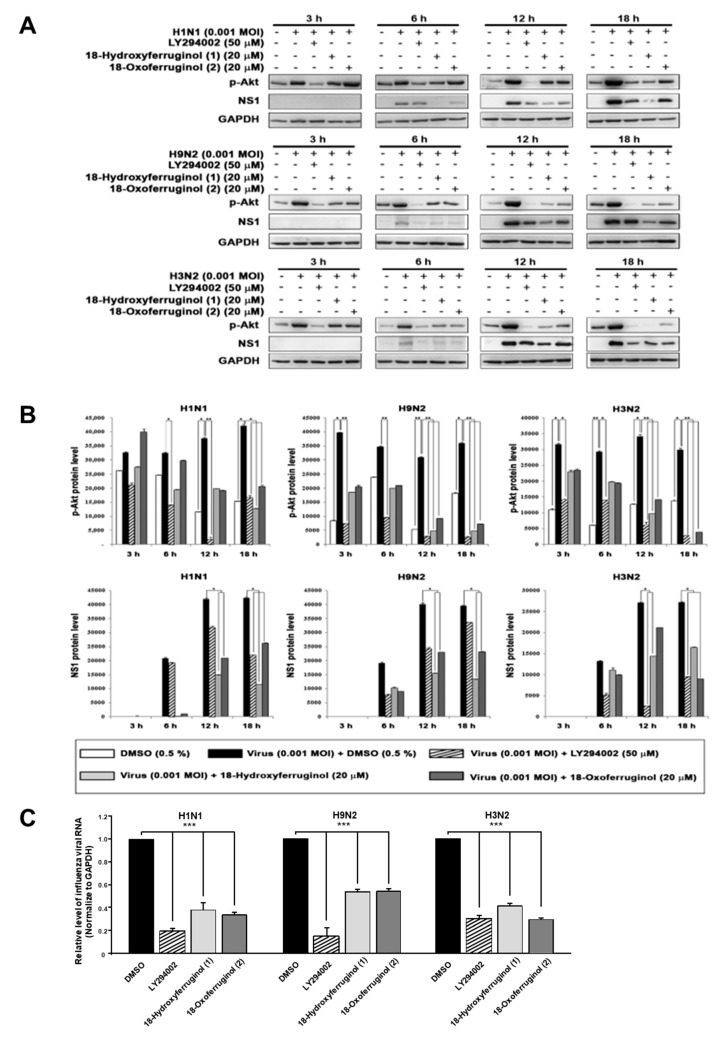
18-Hydroxyferruginol (**1**) and 18-oxoferruginol (**2**) inhibit influenza-virus-induced activation of the PI3K-Akt signaling pathway. (**A**) MDCK cells were infected with H1N1, H9N2, or H3N2 at 0.001 MOI for 1 h and then incubated with 0.5% DMSO, 50 μM LY294002, 20 μM 18-hydroxyferruginol (**1**), or 20 μM 18-oxoferruginol (**2**) for 3, 6, 12, and 18 h. p-Akt and NS1 levels were measured using Western blot analysis. (**B**) The band densities of p-Akt and NS1 proteins was quantified using ImageJ software. Phosphorylated Akt and NS1 protein accumulations were normalized by the GAPDH level. (**C**) After infecting them with influenza viruses, MDCK cells were treated with 50 μM LY294002, 20 μM 18-hydroxyferruginol (**1**), or 20 μM 18-oxoferruginol (**2**). Total RNA was extracted 10 h after influenza virus infection, and the level of intracellular influenza viral RNA (NP gene) was measured. * *p* < 0.05; ** *p* < 0.01; *** *p* < 0.001.

Since the compounds may inhibit some steps after virus replication to reduce viral RNA and protein expression, we hypothesized that the effects of 18-hydroxyferruginol (**1**) and 18-oxoferruginol (**2**) on viral replication may influence some cellular signaling pathways for viral infection. The PI3K-Akt signaling pathway was reported to be required for virus replication, including influenza virus [9,12,13]. In this study, an examination of p-Akt (Serine 473) showed that treatment with compounds **1** and **2** after viral infection decreased p-Akt levels compared with viral-infected/untreated cells (Figure 2A,B). The accumulation of viral proteins, especially NS1, which associates with PI3K, was markedly decreased by compound **1** and **2** treatments (Figure 2A,B). The LY294002 compound was used as a positive control for the inhibition of the PI3K-Akt pathway and viral replication. Moreover, p-Akt was markedly inhibited more strongly by compounds **1** and **2** in the late stage (12 and 18 h) than in the early stage (3 and 6 h) for all viral strains (Figure 2A,B). We found that influenza vRNA levels (NP gene) were reduced 3.3–5.2-fold by LY294002, 2–2.5-fold by compound **1**, and 2–3-fold by compound **2** in all three strains (Figure 2C). These results suggest that the reduction in viral RNA and viral protein levels by compounds **1** and **2** may be due to the inhibition of the PI3K-Akt signaling pathway.

### 3.3. Inhibition of Influenza Ribonucleoprotein (RNP) Nuclear Export by 18-Hydroxyferruginol *(**1**)* and 18-Oxoferruginol *(**2**)*

**Figure 3:**
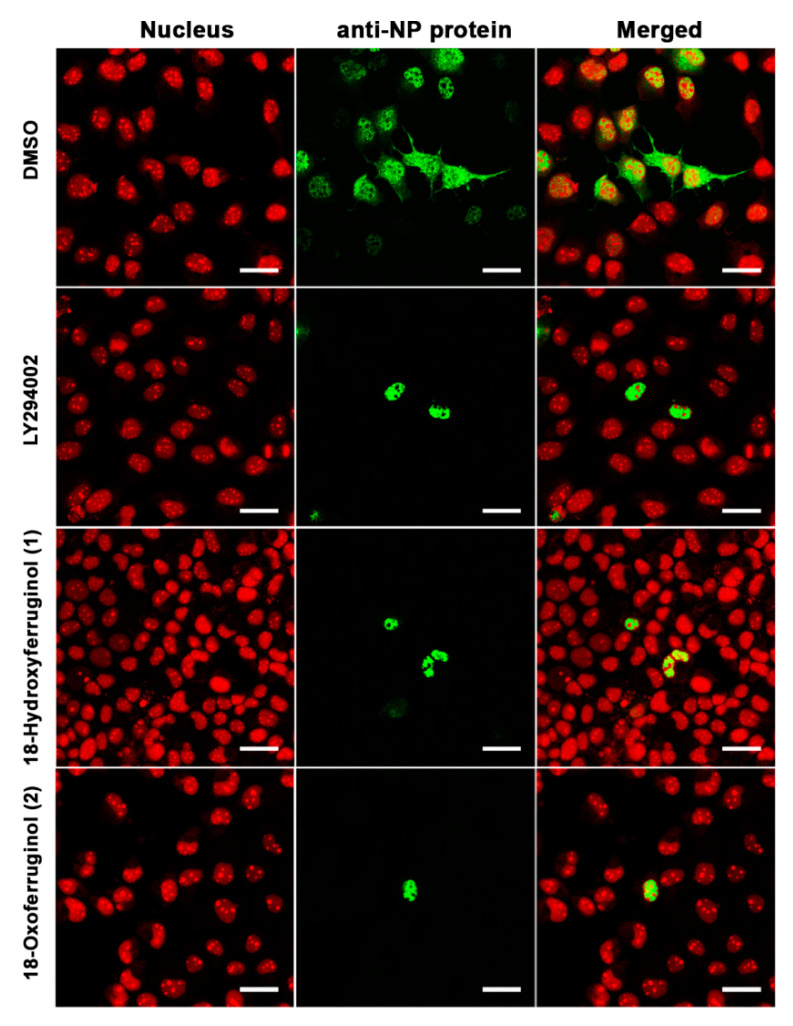
18-Hydroxyferruginol (**1**) and 18-oxoferruginol (**2**) inhibit the export of the influenza virus RNP complex from the nucleus to the cytoplasm. MDCK cells were infected with H1N1 at 0.001 MOI in the presence of 0.5% DMSO, 50 μM LY294002, 20 μM 18-hydroxyferruginol (**1**), or 20 μM 18-oxoferruginol (**2**). After 10 h, the cells were fixed in 4% paraformaldehyde. After blocking, the cells were incubated with the anti-NP antibody (green). Propidium iodide was used as a nuclear counterstain (red). Scale bar = 10 µm.

The inhibition of PI3K-Akt activation by LY294002 led to a reduction in influenza vRNA synthesis and viral protein expression, possibly as a consequence of low NP and M1 protein levels, and viral RNP nuclear export was also suppressed [9]. Therefore, we examined the intracellular trafficking of influenza RNP in virus-infected cells using immunofluorescence. NP was found mainly in the cytoplasm of influenza virus (H1N1)-infected cells, indicating that the majority of the RNP complexes had been exported from the nucleus (Figure 3). However, treatment with compounds **1** and **2** after infection with influenza virus led to a reduction in influenza RNP export from the nucleus to the cytoplasm, indicating that these compounds disturbed viral RNP nucleus-to-cytoplasm export (Figure 3)

### 3.4. Downregulation of Influenza-Virus-Induced ERK Phosphorylation by 18-Hydroxyferruginol *(**1**)* and 18-Oxoferruginol *(**2**)* in MDCK Cells

**Figure 4:**
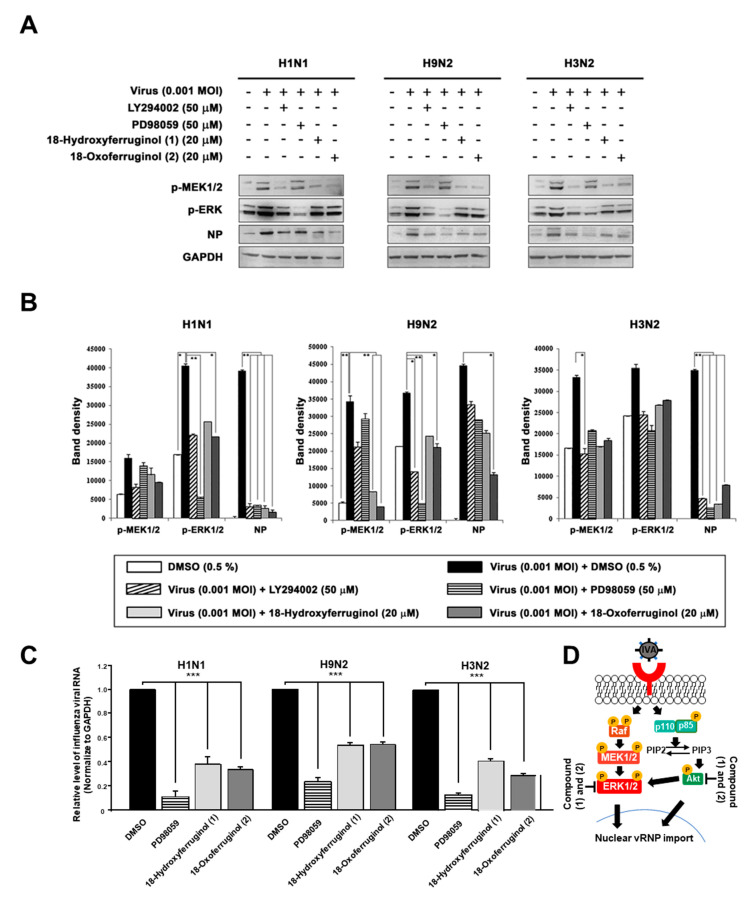
18-Hydroxyferruginol (**1**) and 18-oxoferruginol (**2**) inhibit influenza-virus-induced ERK phosphorylation. MDCK cells were infected with H1N1, H9N2, or H3N2 at 0.001 MOI for 1 h and incubated with DMSO (0.5%), LY294002 (50 μM), PD98059 (50 μM), 18-hydroxyferruginol (**1**) (20 μM), or 18-oxoferruginol (**2**) (20 μM) for 10 h. (**A**) p-MEK1/2, p-ERK, and NP levels were measured by Western blot analysis. (**B**) The band density was quantified by using ImageJ software. Phosphorylated ERK1/2, MEK1/2, and NP protein accumulations were normalized by the GAPDH level. (**C**) After infecting them with influenza viruses, MDCK cells were treated with 50 μM PD98059, 20 μM 18-hydroxyferruginol (**1**), or 20 μM 18-oxoferruginol (**2**). Total RNA was extracted 10 h after influenza virus infection, and the level of intracellular influenza viral RNA (NP gene) was measured. * *p* < 0.05; ** *p* < 0.01; *** *p* < 0.001. (**D**) Schematic depiction of 18-hydroxyferruginol (**1**) and 18-oxoferruginol (**2**) actions on PI3K-Akt and ERK signaling pathways and influenza replication.

Furthermore, ERK activation has been reported to be crucial for the proliferation and nuclear export of influenza viruses [18]. Therefore, we aimed to determine whether compounds **1** and **2** affect ERK activation. The results show that the infection of influenza viruses induced ERK phosphorylation, as expected (Figure 4). Notably, both compounds **1** and **2** inhibited viral-infection-induced ERK phosphorylation. Interestingly, LY294002 strongly inhibited ERK phosphorylation, producing effects similar to those of PD98059 (Figure 4). We also examined the effects of compounds **1** and **2** on the phosphorylation of MEK1/2, an upstream element in the ERK signaling cascade. Infection with influenza viruses induced the phosphorylation of MEK1/2, an effect that was inhibited by compounds **1** and **2** in all strains (Figure 4A). The inhibition of the MEK/ERK signaling pathway by compounds **1** and **2** also resulted in a reduction in the levels of influenza virus NP and thus interfered with virus replication in all strains (Figure 4B). Using qRT-PCR to determine whether the inhibition of ERK activation altered the production of vRNA, we found that influenza vRNA levels were reduced 4.3–9.1-fold by an ERK inhibitor (PD98059) in all three strains (Figure 4C). We show a schematic representation indicating that compounds **1** and **2** inhibited virus replication by blocking the PI3K-Akt and ERK signaling pathways (Figure 4D).

## 4. Discussion

To date, three main types of antiviral agents have been approved by the FDA to treat influenza virus infection. However, these drugs are associated with the emergence of drug resistance and side effects such as nausea and vomiting [25]. Therefore, it is crucial to continue the search for possible replacements for currently used anti-influenza drugs. In a previous study, abietane diterpenoids isolated from *T. nucifera* were reported to have antiviral activity against SARS-CoV 3CL^pro23^. However, the anti-influenza activity and regulatory mechanism of 18-hydroxyferruginol (**1**) and 18-oxoferruginol (**2**) are currently unclear. This is the first study, to our knowledge, to demonstrate that compounds **1** and **2** isolated from *T. nucifera* inhibit the replication cycle of influenza virus in the late stage and block the export of influenza RNP from the nucleus to the cytoplasm [15,26]. Moreover, we defined how compounds **1** and **2** affect the replication of influenza virus, demonstrating that the anti-influenza activity of compounds **1** and **2** is attributable to the inhibition of the PI3K-Akt signaling pathway.

Important in determining the stage at which compounds **1** and **2** exhibit inhibitory activities was the use of time-of-addition assays that focused on three distinct time points: pre-treatment, simultaneous treatment, and post-treatment. In this study, the two compounds exhibited inhibitory effects against influenza virus in the post-treatment assay against all three strains (H1N1, H9N2, and H3N2) (Table 1). Interestingly, after viral infection, influenza vRNA levels were reduced by compounds **1** and **2** predominantly in the late stage (12 and 18 h) of the virus replication cycle rather than in the early stage (3 and 6 h) (Figure 1B). Moreover, compounds **1** or **2** against the three strains showed little NA-inhibitory activity in chemiluminescence-based NAI assays, suggesting that the inhibition of NA is not a major contributor to the antiviral action of these compounds (Table 2). Collectively, these results suggest that compounds **1** and **2** exert their antiviral activity through the inhibition of the viral replication step after virus adsorption rather than by blocking viral entry or viral budding steps.

The antiviral activity of compounds **1** and **2** can be explained by considering the distinctive features of the antiviral mechanism. Like other viral pathogens, influenza viruses hijack factors of the host cell signaling machinery for efficient replication [27,28]. Notably, the NS1 protein of influenza virus interacts with PI3K-Akt, and the interaction is effective for the activation of PI3K-Akt [13,29]. In this study, we found that the phosphorylation of Akt, viral protein synthesis (NS1), and vRNA levels for viral replication were markedly inhibited in the late stage (12 and 18 h) by compounds **1** and **2** and LY294002 (Figure 2). We also found that the nuclear export of viral RNP was impaired in MDCK cells treated with compounds **1** or **2** or LY294002 (Figure 3). Collectively, these results strongly suggest that compounds **1** and **2** exert their anti-influenza activity by inhibiting the PI3K-Akt signaling pathway and blocking viral RNP export from the nucleus, which are important in the viral replication step after virus infection.

The crosstalk between the PI3K pathway and other pathways includes a vast array of other signaling mediators that affect several cell functions [29]. Notably, robust crosstalk between PI3K-Akt and ERK signaling is crucial for the regulation of cell differentiation and survival [30]. It has also been reported that influenza viruses induce ERK activation in both the early and late phases after infection [15,16]. In this study, Western blot analyses revealed that ERK was phosphorylated in virus-infected MDCK cells. On the other hand, compounds **1** and **2** strongly inhibited viral-infection-induced ERK phosphorylation (Figure 4) and substantially reduced vRNA levels (Figure 4C). Interestingly, the inhibition of ERK phosphorylation has been reported to block the export of influenza RNP from the nucleus to the cytoplasm of infected cells [18]. In this study, we found that compounds **1** and **2** markedly decreased the accumulation of NP in virus-infected cells. Therefore, we suggest that the inhibition of PI3K-Akt and ERK signaling blocks the export of influenza RNP from the nucleus to the cytoplasm and subsequently inhibits the replication of influenza viruses.

This study provides the first biochemical evidence that 18-hydroxyferruginol (**1**) and 18-oxoferruginol (**2**) from *T. nucifera* can inhibit influenza virus infection by modulating the PI3K-Akt and ERK signaling pathways. The consequence of these events is the inhibition of viral RNP export from the nucleus and the disruption of influenza virus replication. Therefore, we suggest that 18-hydroxyferruginol (**1**) and 18-oxoferruginol (**2**) can be good candidates for the development of natural therapeutic drugs against seasonal pandemic influenza virus infection via different administration routes.

## Figures and Tables

**Table 1:** Anti-influenza activity of extract and two compounds isolated from *Torreya nucifera* in post-treatment assay.

Extracts or Compounds	CC_50_ (μM) ^a^	A/PR/8/34 (H1N1)		A/Chicken/Korea/MS96/96 (H9N2)		A/Hong Kong/8/68 (H3N2)	
		IC_50_ (μM) ^b^	SI ^c^	IC_50_ (μM) ^b^	SI ^c^	IC_50_ (μM) ^b^	SI ^c^
Olseltamivir	>500	1.35 ± 1.2	>370	<0.7	>714	1.09 ± 0.5	>458
EtOH extract	95.7 ± 1.2 μg/mL	57.9 ± 3.5 μg/mL	1.65	-	-	-	-
18-Hydroxyferruginol (**1**)	35.5 ± 0.4	13.6 ± 1.4	2.61	12.8 ± 0.1	2.77	-	-
18-Oxoferruginol (**2**)	41.2 ± 1.5	18.3 ± 6.5	2.25	10.8 ± 2.2	3.81	29.2 ± 1.9	1.41

^a^ CC_50_: mean (50%) value of cytotoxic concentration. ^b^ IC_50_: mean (50%) value of inhibitory concentration. ^c^ SI: selective index, CC_50_/IC_50_.

**Table 2:** Inhibitory activity of 18-hydroxyferruginol (**1**) and 18-oxoferruginol (**2**) on neuraminidase from influenza A viruses.

Compounds	Neuraminiase IC_50_ (μM) ^a^		
	A/PR/8/34 (H1N1)	A/Chicken/Korea/MS96/96 (H9N2)	A/Hong Kong/8/68 (H3N2)
Olseltamivir	0.011	0.005	0.011
18-Hydroxyferruginol (**1**)	>100	82.2	>100
18-Oxoferruginol (**2**)	>100	>100	>100

^a^ All compounds were examined in a set of duplicated experiments; IC_50_ values of compounds represent the concentration that caused 50% enzyme activity loss.

## Data Availability

The data that support the findings of this study are available upon request from the corresponding author.
